# Immune Reconstitution Inflammatory Syndrome in children

**DOI:** 10.4102/sajr.v21i2.1257

**Published:** 2017-11-14

**Authors:** Nasreen Mahomed, Gary Reubenson

**Affiliations:** 1 Department of Radiology, Faculty of Health Sciences, University of the Witwatersrand, South Africa; 2 Department of Paediatrics & Child Health, Faculty of Health Sciences, University of the Witwatersrand, South Africa

## Abstract

Immune Reconstitution Inflammatory Syndrome (IRIS) refers to a collection of inflammatory disorders, predominantly related to infectious processes that manifest after the initiation of antiretroviral therapy (ART) and can be classified as unmasking or paradoxical. The prevalence of IRIS in children in sub-Saharan Africa is low. Approximately half of all cases are associated with *Mycobacterium tuberculosis*. It may be difficult to distinguish IRIS from tuberculosis and other opportunistic infections radiologically; therefore, radiological findings must be interpreted with clinical and laboratory findings. In this review article, we describe the clinical and radiological manifestations of IRIS in children and provide illustrative radiological examples.

## Introduction

Antiretroviral therapy (ART) has revolutionised the care of HIV-infected children. Compared to untreated children, life expectancy and quality of life are substantially better for children receiving ART.^1,2^ However, like any medication, ART has side effects and potential adverse consequences; the Immune Reconstitution Inflammatory Syndrome (IRIS) is one such adverse consequence.^3,4,5^

IRIS refers to a collection of inflammatory disorders, predominantly related to infectious processes that manifest after initiation of ART. It is usually a self-limiting condition, but on occasion may be severe and rarely can be life-threatening. In most cases, ART can be continued and appropriate antimicrobial therapy is provided for the relevant opportunistic infection.^6^

The immunological changes that occur following initiation of ART are complex,^7,8^ but as the name implies, IRIS occurs when ART-associated improved immune function results in an excessive immune response.^9^ Interestingly, paradoxical worsening of symptoms following initiation of treatment has been described in HIV-uninfected patients and follows a similar improvement in immune function. Paradoxical tuberculosis (TB) reactions are the best known examples of this.^10,11^

## Epidemiology

Early, retrospective studies estimated IRIS incidence as high as 30%; however, more recent evidence suggests a much lower rate of < 10%.^5,6,12,13^ A recent South African study using the International Network for the Study of HIV-associated IRIS criteria reported a paradoxical TB-IRIS incidence of 4.8% among 104 children under 8 years of age.^14^ The incidence of IRIS varies according to the associated micro-organism or underlying condition, the definition of IRIS used, including whether paradoxical or unmasking forms of IRIS or both are included, the region of the world and local incidence of particular opportunistic infections and age of the children. Epidemiological studies on the overall incidence of IRIS and on specific forms of IRIS in children are limited in number and as a result of the factors mentioned above.

## Classification

IRIS is typically classified as either unmasking or paradoxical; however, this distinction may be difficult, as both forms may coexist in the same child and other explanations for the findings should be considered.^15^

Unmasking IRIS refers to clinical features related to a previously undiagnosed, asymptomatic or subclinical infection, whereas paradoxical IRIS is worsening of a previously diagnosed or partially treated condition. Non-infectious forms of IRIS include autoimmune processes and malignancies.^14,15,16,17^

Precise diagnostic criteria for IRIS have been suggested, but consensus on such criteria has not been reached. However, IRIS should be strongly considered when:^9,18^

- ART has been recently initiated, usually within the last 2–6 weeks, but IRIS has been reported many months after initiation.
- There has been a substantial decline in HIV viral load, usually with a significant rise in CD4 count – however, IRIS may occur in the absence of either or both of these.
- Local or systemic inflammatory changes occur related to a previously diagnosed (paradoxical IRIS) or unrecognised (unmasking IRIS) infectious condition – however, IRIS has been reported for non-infectious conditions, including malignancies and autoimmune diseases.
- An alternative explanation for these changes is not identified. This can be very challenging as progression of HIV, new opportunistic infections, drug-resistant pathogens, and medication side effects may present similarly.

## Risk factors

The risk for and severity of IRIS are largely informed by two factors. Firstly, the degree of CD4+ T-cell immune suppression; the lower the pre-treatment CD4 count, the higher the risk of IRIS.^13^ Secondly, the virological and immunological responses to ART; the more rapid the decline in HIV viral load and the faster the rise in CD4 count, the greater the likelihood of developing IRIS.^13,18^ This likely explains the declining incidence in paediatric IRIS as children are being diagnosed earlier and are started on ART soon thereafter, usually before progression to profound immune suppression.^19^

## Clinical manifestations

Clinical manifestations of IRIS are dependent on the implicated pathogen and frequently involve worsening of previously identified manifestations or the onset of new symptoms, similar to those encountered with non-IRIS–related infections with the same pathogen.

*Mycobacterium tuberculosis* (TB) is implicated in approximately half of adults with IRIS.^21^ TB is less commonly implicated in paediatric IRIS, possibly related to the paucibacillary nature of paediatric TB.^14^ Other pathogens commonly implicated are Bacille Calmette–Guérin (BCG) vaccine,^22,23^ cytomegalovirus (CMV)^24^ and non-tuberculous mycobacteria (NTM).^25^ Less common causes of paediatric IRIS include herpes simplex, Kaposi sarcoma (caused by human herpesvirus 8),^26,27^ *Cryptococcus neoformans*,^28^ *Pneumocystis jirovecii*^29^ and parvovirus B19.^30^ This differs from adults, where cryptococcal IRIS^28^ is more likely and BCG-IRIS essentially does not occur. Systemic manifestations include fever, weight loss, fatigue and pain.^20^ Common clinical manifestations of IRIS in children are tabulated in Table 1. In children dermatological manifestations such as seborrhoeic dermatitis and herpes zoster are not uncommon, generally mild and not associated with radiological changes.

**TABLE 1:** Common clinical manifestations of Immune Reconstitution Inflammatory Syndrome.

Pathogen	Clinical features	Comments
TB	Worsening respiratory complaints Symptoms related to airway compression Pleural effusion (new or progression) Lymphadenitis Central nervous system involvement, including focal signs, seizures and meningism	New central nervous system lesions may be asymptomatic Aspirates from lymph nodes commonly culture-negative
BCG	Inflammatory changes and abscess formation at injection site or draining lymph nodes (axillary and cervical) Rarely, evidence of disseminated disease (e.g. hepatosplenomegaly or osteomyelitis)	Localised disease is generally a clinical diagnosis that seldom requires microbiological confirmation
CMV	Pneumonitis Retinitis	Pneumonitis more common than in adults, but retinitis much less common
NTM (e.g. *Mycobacterium avium*)	Lymphadenitis Abdominal complaints	Frequently culture-negative with well-formed granulomas on histology

TB, tuberculosis; BCG, Bacille Calmette–Guérin; CMV, cytomegalovirus; NTM, non-tuberculous mycobacteria.

## Radiological manifestations

### *Mycobacterium tuberculosis*–associated Immune Reconstitution Inflammatory Syndrome

Radiologically it is important to distinguish IRIS from TB or other opportunistic infections, including NTM and CMV. Drug-resistant TB is a clinical and laboratory diagnosis, not a radiological diagnosis and both drug-sensitive and drug-resistant TB can manifest IRIS reactions.^17,31^

**FIGURE 1:**
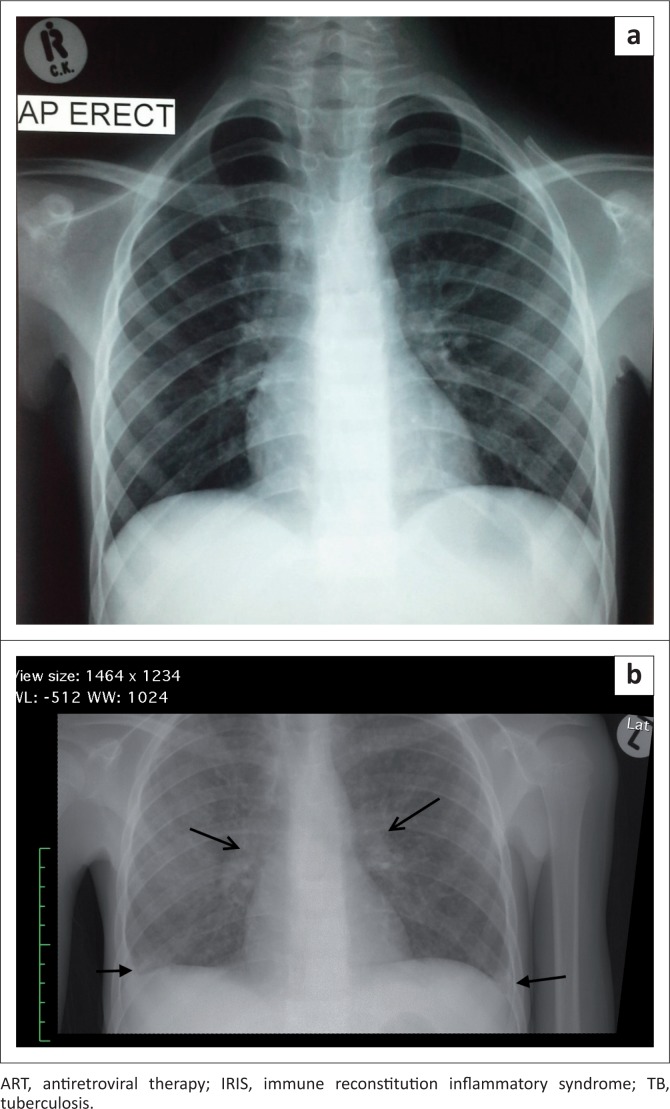
An 11-year-old HIV-infected boy, CD4 count 9 cells/mm^3^ (4%), was initiated on ART. He developed progressive symptoms consistent with disseminated TB and responded well to continuation of ART and standard four-drug TB treatment. Frontal chest radiograph taken at the time of ART initiation (a) demonstrates a normal chest radiograph. Frontal chest radiograph (b) taken 3 weeks after ART initiation demonstrates a diffuse reticular nodular infiltrate with bilateral hilar lymphadenopathy (open arrows) and small pleural effusions (closed arrows). A diagnosis of unmasking TB-IRIS was made.

**FIGURE 2:**
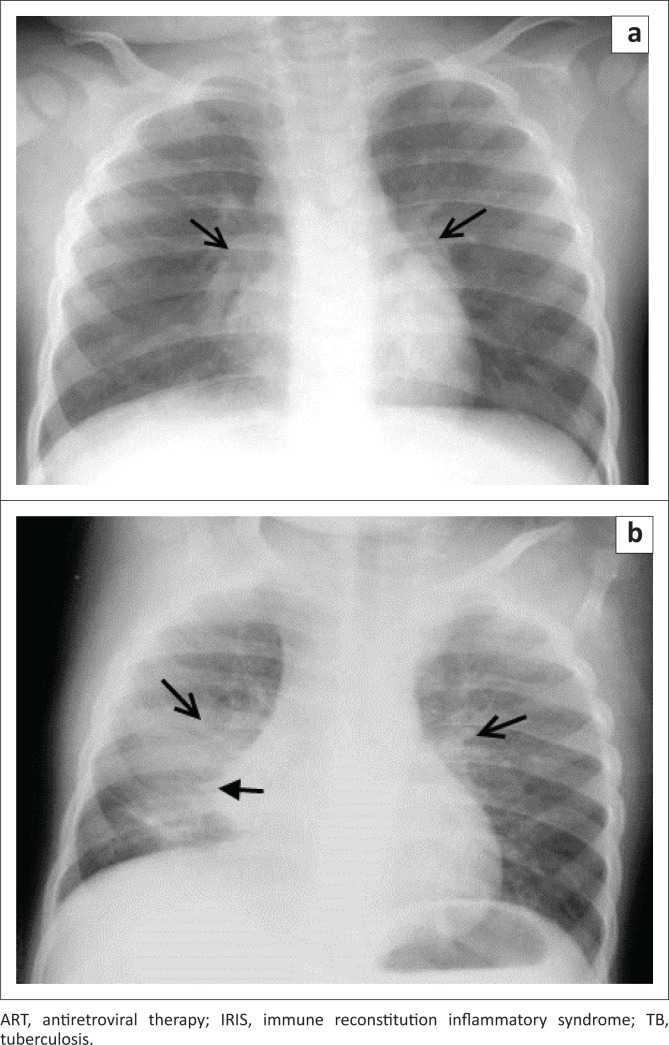
A 3-year-old HIV-infected girl initiated on ART 2 weeks after starting TB treatment. CD4 count was not available. Frontal chest radiograph taken at the time of ART initiation: (a) demonstrates bilateral hilar lymphadenopathy (open arrows). Chest radiograph taken 2 weeks after ART initiation; (b) demonstrates right middle lobe consolidation and collapse (closed arrow), with worsening hilar lymphadenopathy (open arrows). In combination with the clinical, radiological and laboratory findings, a diagnosis of paradoxical TB-IRIS was made.

**FIGURE 3:**
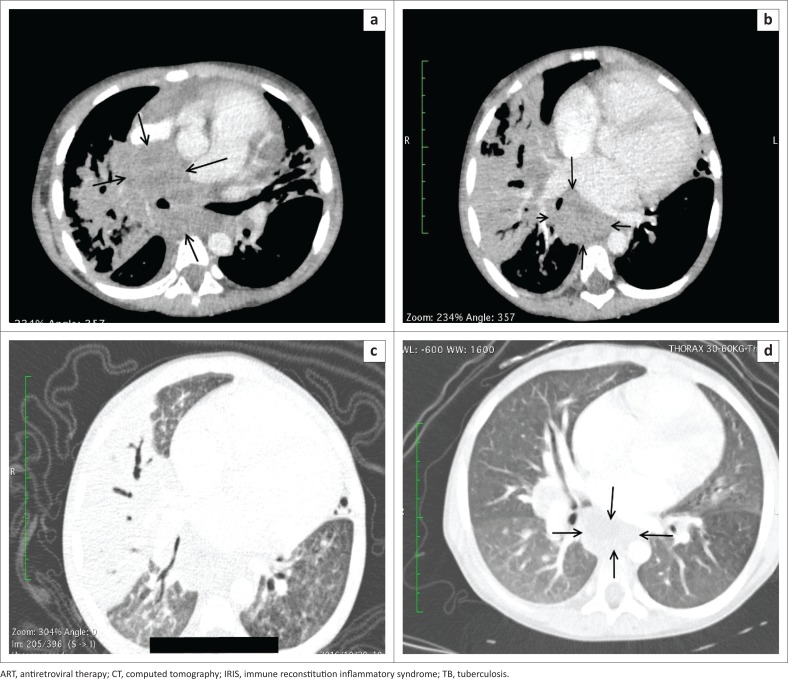
A 4-year-old HIV-infected boy living with an adult diagnosed with pulmonary TB was initiated on ART. Three months later, he developed symptoms of pulmonary TB, which was microbiologically confirmed (Unmasking IRIS). He was initiated on appropriate TB treatment and initially improved; 3 weeks later he presented with fever and worsening respiratory symptoms and signs (Paradoxical IRIS). Computed tomography of the chest, axial, soft tissue window (a and b) demonstrates mediastinal and hilar lymph nodes (arrows) encasing the bronchi bilaterally. There is also posterior mediastinal involvement with anterior displacement of the left atrium and a small right pleural effusion; (c) axial lung window demonstrates dense right middle lobe and apical segment of right lower lobe consolidation with lingular consolidation. A diagnosis of paradoxical and unmasking TB-IRIS was made. ART and TB treatment were continued and corticosteroids were administered; he demonstrated a good clinical response and is currently well; (d) axial CT chest axial lung window performed 6 months later demonstrates resolution of the consolidation, with residual lymphadenopathy (open arrows).

The most common radiological manifestations of TB-IRIS occur within the chest and include new or worsening hilar or mediastinal lymphadenopathy, which may cause tracheo-bronchial compression. Other common chest radiological findings include worsening or new air space consolidation, pleural effusions, reticular infiltrates or nodular infiltrates (Figures 1–3).^14,16,17,31^

Abdominal manifestations of TB-IRIS include ascites, abdominal lymphadenopathy, and splenic micro-abscesses.^16^ Central nervous system (CNS) manifestations are uncommon and include TB meningitis, which may cause obstructive hydrocephalus, or associated tuberculomas.^16^

### Bacille Calmette–Guérin–associated Immune Reconstitution Inflammatory Syndrome

**FIGURE 4:**
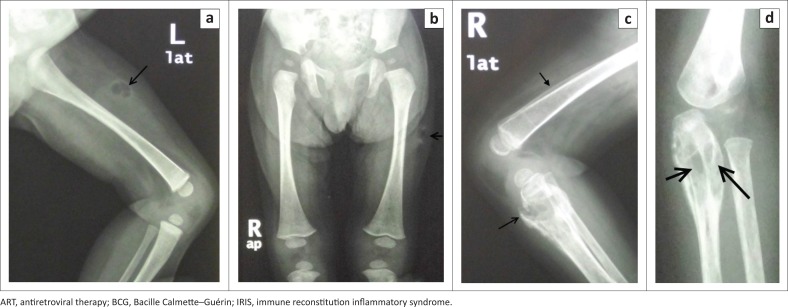
A 9-month-old HIV-infected girl, with CD4 count 9 cells/mm^3^ (1.53%), was admitted with severe malnutrition, right axillary and left thigh abscesses, generalised lymphadenopathy, hepatosplenomegaly and clinical evidence of HIV encephalopathy. ART was initiated with subsequent worsening of the thigh abscess. An aspiration was performed on the left thigh abscess which cultured *Mycobacterium bovis* BCG, confirmed using molecular testing. Appropriate antimycobacterial treatment was provided: (a) (lateral) and (b) (AP), radiograph of the lower limbs demonstrate an air containing abscess in the upper left lateral thigh soft tissues (open arrow) and generalised left lower limb soft tissue swelling. No periosteal reaction was demonstrated in visualised bones. She was readmitted a year later, still on antimycobacterial treatment with soft tissue abscesses in the right thigh, forehead, abdominal wall and left forearm; (c) (left lateral knee) demonstrates osteomyelitis, with lytic lesions within the proximal tibial metaphysis and a cortical break along the anterosuperior tibial margin (open arrow). There is periosteal reaction along the tibia and distal femur (closed arrow) with generalised soft tissue oedema; (d) (left AP forearm) demonstrates another focus of osteomyelitis with lytic lesions in the proximal metadiaphysis of the ulna (open arrows), associated periosteal reaction and osteopenia of the left ulna and radius. Bone aspiration again confirmed *M. bovis* BCG, now resistant to first-line antimycobacterial agents, necessitating surgical drainage; however, the patient did not survive. A diagnosis of BCG-IRIS was made.

BCG-associated IRIS is well recognised^16^ and in some settings is the most commonly identified cause.^16,22^ It usually manifests with inflammatory changes at the injection site and/or within the ipsilateral draining lymph nodes. Chest radiographic changes are uncommon and may be indistinguishable from TB.^22^ Unusual manifestations of BCG-IRIS have been reported (Figure 4).

### *Mycobacterium avium* complex Immune Reconstitution Inflammatory Syndrome

*Mycobacterium avium* complex (MAC)–associated IRIS manifests radiologically as necrotic lymph nodes identified on ultrasound, computed tomography or magnetic resonance imaging. These may be indistinguishable from other mycobacterial infections. The diagnosis of MAC may be made from tissue culture;^16^ however, cultures are commonly negative. Therefore, a high level of clinical suspicion is required.

Radiologically, it may be difficult to distinguish IRIS from TB and other opportunistic infections so radiological findings must be interpreted in combination with clinical and laboratory findings.^16,17,31^

## Non-radiological investigation

Investigation is largely informed by the particular infection under consideration. Measurement of viral load and CD4 counts are helpful, particularly when poor adherence to therapy is suspected. A significant decline in viral load from baseline, as well as a substantial increase in CD4 count, is suggestive of IRIS, but not essential for making the diagnosis.^9,18^ Of note, children with suspected local or regional BCG-IRIS can generally be diagnosed based on clinical manifestations and microbiological confirmation using lymph node biopsy or fine-needle aspiration is seldom warranted unless alternative diagnoses are under consideration. If samples are submitted from patients with suspected BCG-related disease, it is vital to indicate this on the laboratory request form so that appropriate testing to differentiate TB from BCG can be performed.

## Clinical management

The most important components of effective management are to treat the IRIS-related infection using the same modalities as for non-IRIS–related infections with that pathogen. Because IRIS is frequently mild and self-limiting, in most patients continuation of ART is to be encouraged.^6^ In patients with severe or life-threatening IRIS, including airway compression by hilar or mediastinal lymph nodes or expansile CNS mass lesions, it may rarely be appropriate to interrupt ART until their condition stabilises, whereafter ART can be safely restarted.^6^ Stopping ART is not to be taken lightly and when being considered should involve discussion with a specialist or subspecialist.

In children with pain and discomfort from BCG-IRIS lymphadenitis, consideration should be given to needle aspiration of fluctuant areas to relieve symptoms. Such aspiration may need to be repeated.^22^ In addition to continuation of ART and treating the implicated pathogen, patients with IRIS may benefit from anti-inflammatory medicines. In mild and moderate cases, non-steroidal anti-inflammatory drugs (NSAIDs) have been used to provide symptomatic relief and may hasten IRIS resolution. In more severe cases, corticosteroids may be required for a number of weeks, whereafter they can be tapered and stopped.^6^ The additional immune suppression related to corticosteroid use is a concern in such patients and specialist input is advised before their commencement.

## Prevention

If HIV is diagnosed early and ART initiated soon thereafter, IRIS is unlikely.^23^ Recent improvements in early infant diagnosis of HIV followed by almost immediate initiation of ART have resulted in IRIS becoming uncommon.^23^

Furthermore, it is important to consider opportunistic infection prior to ART initiation as initiating treatment for them first may reduce the risk of IRIS developing. This is particularly true for CNS TB and cryptococcal meningitis where ART initiation should be delayed during the initial weeks of treatment in order to reduce the risk of IRIS. Tuberculous mass lesions (tuberculomas) have been associated with life-threatening CNS-IRIS.^13,32^ In most patients with TB, ART can be safely initiated about 2 weeks after TB treatment has commenced; however, extrapolating from experience in adults with HIV-associated cryptococcal meningitis, ART initiation should be delayed by at least 6–8 weeks.^33^ Routine prophylactic use of NSAIDs or corticosteroids to reduce the risk of IRIS is not recommended, rather patients at high risk for IRIS should be closely monitored for new or worsening symptoms so that IRIS can be timeously identified and treated.

## Conclusion

While ART has revolutionised the care of HIV-infected children, ART has side effects and potential adverse consequences which include IRIS. IRIS is typically classified as either unmasking or paradoxical; however, this distinction may be difficult, as both forms may coexist in the same child and other explanations for the clinical findings should be considered. Approximately half of all IRIS (adult and paediatric) cases are associated with *M. tuberculosis*. The commonest radiological manifestations of TB-IRIS include new or worsening hilar or mediastinal lymphadenopathy, worsening or new air space consolidation, pleural effusions, reticular infiltrates or nodular infiltrates. It may be difficult radiologically to distinguish IRIS in children from TB and other opportunistic infections so radiological findings must be interpreted in combination with clinical and laboratory findings.
